# Excited-State Dynamics of Melamine and Its Lysine Derivative Investigated by Femtosecond Transient Absorption Spectroscopy

**DOI:** 10.3390/molecules21121645

**Published:** 2016-11-30

**Authors:** Yuyuan Zhang, Ashley A. Beckstead, Yuesong Hu, Xijun Piao, Dennis Bong, Bern Kohler

**Affiliations:** 1 Department of Chemistry and Biochemistry, Montana State University, Bozeman, MT 59717, USA; yuyuan.zhang@montana.edu (Y.Z.); ashleya1117@gmail.com (A.A.B.); huys13@lzu.edu.cn (Y.H.); 2 Department of Chemistry and Biochemistry, The Ohio State University, 100 West 18th Avenue, Columbus, OH 43210, USA; piao.6@osu.edu

**Keywords:** *s*-triazines, prebiotic molecules, UV photostability, time-resolved vibrational spectroscopy

## Abstract

Melamine may have been an important prebiotic information carrier, but its excited-state dynamics, which determine its stability under UV radiation, have never been characterized. The ability of melamine to withstand the strong UV radiation present on the surface of the early Earth is likely to have affected its abundance in the primordial soup. Here, we studied the excited-state dynamics of melamine (a proto-nucleobase) and its lysine derivative (a proto-nucleoside) using the transient absorption technique with a UV pump, and UV and infrared probe pulses. For melamine, the excited-state population decays by internal conversion with a lifetime of 13 ps without coupling significantly to any photochemical channels. The excited-state lifetime of the lysine derivative is slightly longer (18 ps), but the dominant deactivation pathway is otherwise the same as for melamine. In both cases, the vast majority of excited molecules return to the electronic ground state on the aforementioned time scales, but a minor population is trapped in a long-lived triplet state.

## 1. Introduction

**Figure 1:**
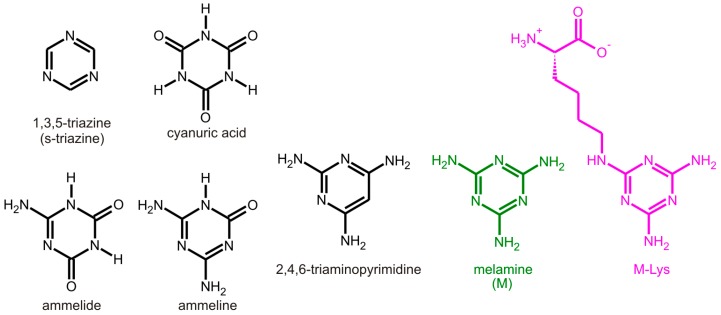
Triazine-based prebiotic recognition elements. The molecules studied here are drawn in color.

The 1,3,5-triazines, or *s*-triazines, represent an important class of prebiotic molecules that may have been building blocks of the primitive informational polymers that preceded RNA. Along with some amino acids and pyrimidines, several *s*-triazines, i.e., melamine, ammeline, ammelide, and cyanuric acid (structures shown in Figure 1), can be synthesized in an icy urea solution under a reductive N_2_/H_2_/CH_4_ atmosphere [1]. It is known that melamine and cyanuric acid form a supramolecular assembly via hydrogen bonding [2], demonstrating the molecular recognition ability of *s*-triazines. Indeed, oligodipeptides tagged with diamino-triazine derivatives form duplex hybrids with oligo-T/U DNA or RNA [3]. More interestingly, mono-derivatized triaminotriazine, or melamine, has two thymine recognition faces. As a consequence, bifacial peptide nucleic acid (bPNA), which displays melamine on peptide side chains, can dock two oligothymidine strands to form a highly stable triplex hybrid with high selectivity over other oligonucleotides [4,5].

The thermal stability of *s*-triazines in different solvents and pH conditions has been studied [6], but their UV hardiness, or intrinsic stability against UV radiation, has not been examined. UV hardiness is an important but often overlooked criterion for selecting possible forerunners of the DNA and RNA nucleobases [7]. It is widely accepted that there was significantly higher solar UV flux down to wavelengths as short as 200 nm near the early Earth’s surface due to the lack of ozone shielding [8,9]. Under these conditions, molecules possessing recognition properties, but prone to degradation upon UV irradiation, were unlikely to accumulate to high enough concentrations to form polymers [7]. Modern-day canonical nucleobases are endowed with a high degree of intrinsic protection against UV radiation. Thus, femtosecond transient absorption (fs-TA) experiments on nucleosides in aqueous solution reveal subpicosecond excited-state lifetimes due to extremely efficient internal conversion [10,11,12], which outcompetes slower photochemical channels such as photoionization, photodissociation, and ring-opening reactions. Ultrashort excited-state lifetimes also greatly reduce the possibility of bimolecular reactions and energy transfer, even in molecularly crowded environments such as those possibly existing in a primordial soup or a proto-cell [7].

Recent fs-TA experiments by Brister et al. [13] indicate that 2,4,6-triaminopyrimidine (Figure 1), a possible proto-nucleobase that is similar to melamine (2,4,6-triamino-1,3,5-triazine), has a subpicosecond ^1^ππ* state lifetime and a ^1^nπ* state lifetime of several ps. Here, we report on the excited-state dynamics of melamine in neutral aqueous solution using fs-TA and time-resolved infrared (TRIR) spectroscopy. The vast majority of excited states decay on a time scale of 13 ps. We also investigated the excited-state dynamics of the lysine derivative of melamine in which the lysine moiety is linked at one of the three amino groups (Figure 1). Mittapalli et al. proposed that amino acids might have been the building blocks of the backbone of prebiotic informational oligomers instead of the modern-day phosphoribose backbone of DNA and RNA [3,14]. The lysine derivative of melamine, a proto-nucleoside, has a similar excited-state lifetime to the melamine chromophore. Our results show that chemically modifying the amino side group of melamine with a non-chromophoric moiety does not significantly alter its excited-state dynamics.

## 2. Results and Discussion

### 2.1. Steady-State Spectra

**Figure 2:**
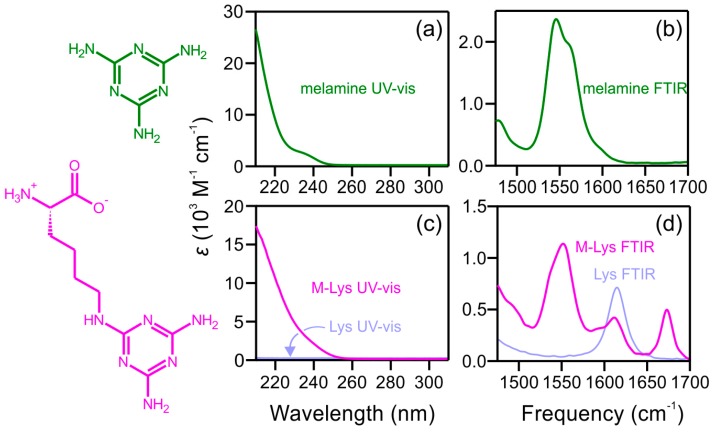
UV-visible absorption spectra of melamine (**a**) and M-Lys (**c**) in neutral D_2_O solution. FTIR spectra for melamine (**b**) and M-Lys (**d**) in neutral D_2_O solution. The UV-visible and FTIR spectra of lysine (light purple) taken under the same experimental conditions are also included. The extinction coefficient (*ε*) is calculated from *ε* = *A*/(*L*·*c*), where *A* is the measured absorbance, *L* is the optical path length (100 μm) and *c* is the concentration of the sample (10 mM).

The p*K*_a_ of melamine is 5.0 [15,16]. The aromatic ring is protonated at low pH, whereas the molecule is uncharged at neutral and basic conditions. The UV-visible absorption spectrum of melamine in neutral D_2_O solution is displayed in Figure 2a. Contrary to the DNA/RNA bases, which absorb strongly at 260 nm, the electronic absorption spectrum of melamine peaks at a much shorter wavelength. As described for the alternative nucleobase urazole [17], shifting the absorption band to a shorter wavelength may reduce the rate of excitation by longer-wavelength UV photons, although possibly at the cost of greater excitation of the nonaromatic backbone, which is normally shielded from excitation by the nucleobases.

The absorption spectrum rises monotonically from 260 nm to 210 nm, and a weak shoulder is seen at 240 nm. This shoulder was assigned by Hirt and Salley to a ^1^ππ* transition, a transition that is symmetry-forbidden in melamine (D_3h_ point group, neglecting the amino protons) [18]. Dewar and Paoloni argued that absorption at 240 nm is due to an electronically forbidden, but vibrationally allowed, ^1^ππ* transition and a ^1^nπ* transition located at approximately the same energy [19]. The extinction coefficient (*ε*) at 240 nm is approximately 1400 M^−1^·cm^−1^, according to our measured absorbance. This value is slightly larger than the one reported in Reference [18] (*ε* = 1100 M^−1^·cm^−1^ at 235 nm), but the latter value was corrected for overlap with the adjoining, short-wavelength band. An extremely weak peak at approximately 300 nm has also been reported and assigned to a spin-forbidden triplet transition [20], but this peak is not evident at our concentration and optical path length.

Preliminary density functional theory (DFT) calculations indicate that the minimum-energy ground-state geometry of gas-phase melamine is virtually planar based on B3LYP/6-31G(d,p) optimization. Time-dependent density functional theory (TD-DFT) calculations using several different functionals and semi-empirical calculations all predict that the lowest-energy ^1^ππ* transition is wholly forbidden. A ^1^nπ* state transition is calculated to have a reasonable oscillator strength, and is proposed to contribute to the 240 nm shoulder seen in Figure 2b. However, experimental evidence suggests that a ^1^ππ* transition also contributes to this feature [19]. Overall, our preliminary calculations support a picture of overlapping and weakly allowed ^1^ππ* and ^1^nπ* transitions. This agrees with studies from the 1950s on melamine [18,19] as well as with more recent electronic structure calculations of the parent molecule *s*-triazine [21,22].

The FTIR spectrum of melamine in the double-bond stretching region in neutral D_2_O solution is shown in Figure 2b. A very strong vibrational band centered at 1545 cm^−1^ and a weaker shoulder at 1560 cm^−1^ are observed. Prominent transitions have also been observed in the 1500–1600 cm^−1^ region for deuterated melamine in an Argon matrix at 10 K [23] and in room-temperature crystals [24]. Although the solvation environments in these experiments differ dramatically from ours, the band positions are within 30 cm^−1^ to those observed in aqueous solution at room temperature. These bands resemble ones seen for the canonical nucleobases between 1550 and 1650 cm^−1^ that are assigned to ring in-plane vibration modes [25,26].

The UV-visible and FTIR spectra of the lysine derivative of melamine (M-Lys) are shown in Figure 2c,d, respectively. Lysine absorbs negligibly at wavelengths longer than 200 nm, but it nevertheless causes the absorption spectrum of M-Lys to change in appearance compared to that of melamine. In particular, the extinction coefficient of the shoulder increases, making it blend with the higher-energy transition. This change is ascribed to breaking of the trigonal symmetry by the lysine substituent, causing the lowest-energy ^1^ππ* state to increase in oscillator strength. A similar effect has been observed when substituting an amino proton with an alkyl or alkylol group [18]. 

The positions of the ring in-plane deformation bands of M-Lys are close to those of unsubstituted melamine, but the relative intensities have changed. The higher frequency band is stronger than the lower frequency one. It is unclear why the intensity of this double band is only half as strong as that of unsubstituted melamine. In addition, a band centered at 1612 cm^−1^ which is assigned to the C=O stretch of lysine is observed in the FTIR spectrum of M-Lys. As the lysine derivative is a trifluoroacetic salt produced from the trifluoroacetic acid cleavage of Boc-Lys(Mel), the highest-frequency band at 1672 cm^−1^ may be due to the carbonyl stretch of trifluoroacetate (see Section 3.3).

### 2.2. Excited-State Dynamics

#### 2.2.1. Melamine (M)

**Figure 3:**
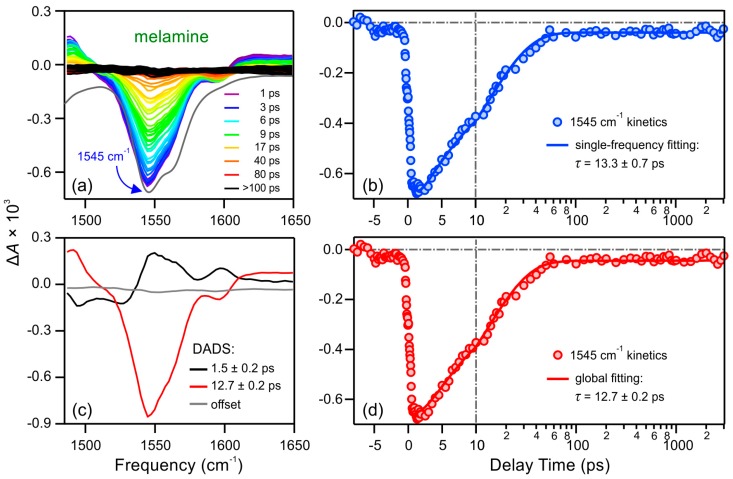
(**a**) TRIR spectra of melamine in neutral D_2_O solution recorded from 1 ps (**purple**) to 3 ns (**black**) after 240 nm excitation. The inverted FTIR spectrum (**gray**) is shown for comparison. The blue curved arrow points to the frequency of the kinetic trace presented in panel (**b**); (**b**) Bleach recovery kinetic trace at 1545 cm^−1^. The blue points are experimental data. The solid blue line is the best-fit curve $A_{1}e^{-t/\tau_{1}}+A_{2}e^{-t/\tau_{2}}+A_{3}$; (**c**) Decay-associated difference spectra (DADS) obtained from global fitting; (**d**) Bleach recovery kinetics trace at 1545 cm^−1^ overlaid with the best-fit curve obtained from global fitting. The vertical dash-dotted lines mark the linear-logarithmic break in the time axis. The time constants obtained from single-frequency and global fits are indicated together with 2σ uncertainties.

Melamine in neutral D_2_O solution was excited at 240 nm, and the ensuing dynamics were probed by broadband mid-IR pulses (see Section 3.1 for details). At this excitation wavelength, both ^1^nπ* and ^1^ππ* states may be populated as discussed in the previous section. The vibrational spectra recorded from 1 ps to 3 ns after excitation are displayed in Figure 3a. The negative peaks, which are in excellent agreement with ones in the inverted FTIR spectrum, are due to ground-state bleaching (GSB), i.e., depletion of the ground-state population due to photoexcitation. The positive signals originate from excited-state absorption (ESA) or absorption by other transient species. All TRIR signals disappear by approximately 80 ps, leaving a featureless offset across the spectral region (black traces in Figure 3a). This negative offset is likely due in part to solvent heating induced by each pump pulse [27]. The strongest GSB signal at 1545 cm^−1^ exhibits a slight buildup to a maximum at approximately 2 ps, and then decays with a time constant of 13.3 ± 0.7 ps (Figure 3b). Globally fitting the two-dimensional data in the spectral and temporal domains gives a similar picture. The majority of the TRIR signal decays monoexponentially with a time constant of 12.7 ± 0.2 ps, but a weak positive component is required to fit the data at times shorter than 2 ps, as shown in Figure 3c,d. The amplitude of the fast component, which could be due to the weak background signal from the CaF_2_ windows or to a rapidly decaying excited state, is considerably weaker than the 13 ps component, as can be seen in Figure 3c. Our focus below is on the dominant decay with a lifetime of ~13 ps.

Our data indicate that the thermally equilibrated ground state is repopulated on a time scale of 13 ps. Crudely, the time constant for GSB recovery is the greater of the excited-state lifetime and the time constant characteristic of vibrational cooling (VC) on the ground state following ultrafast internal conversion (UIC). The 13 ps time constant is too slow for VC in D_2_O as typical VC times for UV-excited canonical DNA nucleotides in D_2_O are 2–5 ps [28]. Furthermore, the VC of nucleotides is facilitated by direct energy transfer from the high-frequency stretching modes such as N–H(D) and O–H(D) of the chromophore (nucleobases) to the high-frequency O–H(D) stretching modes and bending overtones of water [29]. Melamine possesses more hydrogen bond donors (six N–H groups) than any of the canonical nucleobases, suggesting that VC should proceed faster than 2 ps. The much longer observed decay thus suggests that the lifetime of the excited state is 13 ps.

The transient absorption experiment using 240 nm pump and 350 nm probe pulses further supports our proposal. The 350 nm probe (3.54 eV) has much lower energy compared to the onset of significant electronic absorption near 250 nm (4.96 eV), and thus is expected to monitor ESA without the complication of hot ground-state absorption. Although a low-lying singlet-triplet transition is responsible for a very weak band at ~300 nm, as discussed in the previous section, the extinction coefficient of this band is low (estimated to be on the order of 1 M^−1^·cm^−1^ [20]) and it can be neglected.

**Figure 4:**
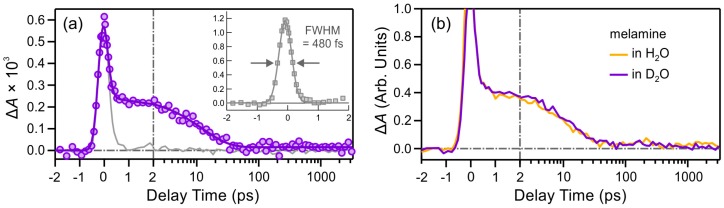
(**a**) Excited-state decay of melamine in neutral D_2_O solution probed at 350 nm after 240 nm excitation. The purple points are experimental data. The solid line is the best-fit curve $A_{0}\delta \left(t\right)+A_{1}e^{-t/\tau_{1}}+A_{2}$, see text. The solid gray line is the instrument response function obtained from pure D_2_O. The fwhm of this signal is 480 fs (inset); (**b**) Excited-state decay of melamine in neutral H_2_O (**orange**) and D_2_O (**purple**) solutions. The solid lines are experimental data.

The kinetic trace monitored at 350 nm is presented in Figure 4a. A delta function (%*A*_0_ = 62% of total amplitude), a monoexponential decay (*τ*_1_) with a lifetime of 12 ± 2 ps (%*A*_1_ = 36%), and a small offset (%*A*_2_ = 2%), convoluted with the instrument response function (IRF), which has a full width at half maximum (fwhm) of 480 fs, are needed to fit the experimental data. The delta function component could be a rapid decay by melamine that is too fast to be resolved by our instrument, but we note that a similar decay is observed in a solvent-only scan (gray trace in Figure 4a). Significantly, this fast component is not seen in the TRIR GSB measurements. For this reason, and also because the *τ*_1_ lifetime measured at 350 nm is in excellent agreement with the lifetime measured in GSB experiments, the fast decay around time zero is most likely due to two-photon (one pump + one probe photon) absorption by water when the pump and probe pulses overlap in the sample. Note that at longer delay times there is no background due to the two-photon (i.e., two pump photons) ionization of water (gray trace in Figure 4a), so the correction for a solvated electron signal described in previous publications [30,31] is not required. 

Next we consider the origin of the excited state that lasts approximately 13 ps. Sequential experiments performed in H_2_O and D_2_O indicate a negligible kinetic isotope effect (Figure 4b), thereby ruling out the possibility of intramolecular or intermolecular proton transfer. This also rules out the possibility of photodissociation whereby melamine ejects an H atom from one of the three amino groups via homolytic bond fission. Earlier, the N–H bond fission in the amino group of 9H-adenine was shown to not be operative even at 220 nm [32].

In light of uncertainty about the nature of the initial excited state or states populated by the pump pulse, it is difficult to provide a fully comprehensive picture of excited-state deactivation. Prior studies discussed above make it plausible that the lowest singlet state could be an nπ* state. Although such a state could possibly be a long-lived trap for the excited-state population, our results clearly indicate that most excited states return to the electronic ground state on a time scale of 13 ps. Previously, nπ* excited states with lifetimes of several tens of ps were observed in the canonical pyrimidine nucleobases [33].

From the GSB signal in the TRIR spectra, it is clear that the vast majority of the excited-state decay occurs in 13 ps. However, given the maximum GSB signal of −7 × 10^−4^ and a noise floor of 5 × 10^−5^ (Figure 3), a long-lived excited state that is weakly populated could escape detection. Indeed, an offset that is 2% of the total amplitude is required to satisfactorily fit the 240 nm pump/350 nm probe decay trace (Figure 4), suggesting that some of the initially excited states are trapped to a long-lived state that persists for more than a nanosecond. This long-lived state is proposed to be a triplet state. Interestingly, Yang et al. described an emission at ~370 nm in room-temperature solution that decays on time scales greater than 10^−8^ s [34]. The large shift between the absorption and emission maximum and the long-lasting emission suggest the presence of triplet states.

#### 2.2.2. Lysine Derivative of Melamine (M-Lys)

**Figure 5:**
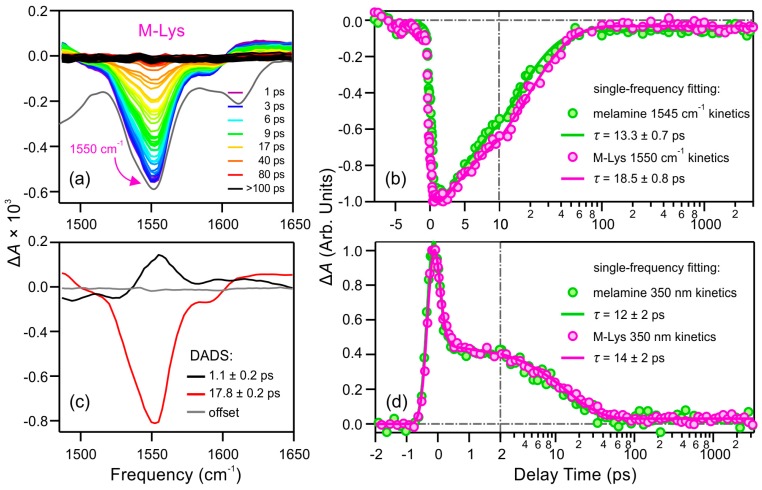
(**a**) TRIR spectra of M-Lys in neutral D_2_O solution recorded from 1 ps (**purple**) to 3 ns (**black**) after 240 nm excitation. The inverted FTIR spectrum (**gray**) is shown for comparison. The magenta curved arrow points to the frequency of the kinetic traces presented in panel (**b**); (**b**) Normalized bleach recovery kinetic trace for M-Lys (magenta) and the unsubstituted melamine (green); (**c**) Decay-associated difference spectra (DADS) for M-Lys obtained from global fitting; (**d**) Normalized excited-state decay of M-Lys (magenta) and the unsubstituted melamine in neutral D_2_O solution probed at 350 nm after 240 nm excitation. The points are normalized experimental data. The solid lines are the best-fit curves (see caption for Figure 3 and Figure 4). The original data (without normalization) for the unsubstituted melamine have been shown in Figure 3b and Figure 4a. The vertical dash-dotted lines mark the linear-logarithmic break. The time constants obtained from single-frequency and global fitting are included. The uncertainties reported are 2σ.

The TRIR spectra of M-Lys in neutral D_2_O solution recorded from 1 ps to 3 ns after 240 nm excitation are displayed in Figure 5a. Peaks in the inverted FTIR spectrum align well with negative peaks in the TRIR signals, with the exception of the FTIR band at 1612 cm^−1^, which has no counterpart in the time-resolved spectra. This band is assigned to the carbonyl stretch of lysine, and the absence of bleaching at this frequency indicates that the melamine chromophore is excited, not the lysine side chain. Because lysine cannot be directly excited to any significant extent at 240 nm, the lack of a TRIR signal from lysine indicates that excitation energy is unable to reach the carbonyl through the aliphatic chain. West et al. have reported vibrational energy transfer from thymine to phosphoribose in UV-excited thymidine [35]. This transfer stems from UIC converting most of the photon energy (265 nm = 4.68 eV) to vibrational energy in the chromophore. In the case of M-Lys, most of the photon energy is trapped in the ^1^ππ* or ^1^nπ* excited state for 13 ps, eliminating the possibility of vibrational energy transfer to the lysine carbonyl group within this time.

Figure 5b shows the kinetics of the strongest GSB signal of M-Lys. Compared to melamine, the recovery time of the UV-excited M-Lys is slightly slower. Global fitting of the TRIR data gives similar spectra as the substituted melamine (compare Figure 5c to Figure 3c), but also reveals a slower time constant, which is in excellent agreement with the single-frequency fitting shown in Figure 5b. This suggests that the excited-state lifetime of M-Lys is slower than that of the unsubstituted melamine. However, kinetics monitored at 350 nm show a smaller difference in lifetimes than is seen in the TRIR measurements.

Hypoxanthine (another possible prebiotic recognition element [36]), its nucleoside inosine, and its nucleotide inosine 5′-monophosphate deactivate via UIC on a time scale of 100 fs following UV excitation [37]. There is no observable difference in the ^1^ππ* state lifetimes of the three molecules [37], indicating that the ribose and phosphoribose moiety do not dramatically change the barrier-free path connecting the Franck-Condon region and the S_1_/S_0_ conical intersection. On the other hand, three- to six-fold increases in the ^1^nπ* lifetimes have been observed when going from canonical pyrimidines (C, T and U) to their nucleotides, while the ^1^ππ* state lifetimes remain largely unaffected [33]. The change in the excited-state lifetime of melamine upon lysine substitution thus resembles the behavior of the canonical pyrimidines upon incorporation of a phosphoribose group. It is interesting to note that *s*-triazines are more structurally similar to pyrimidines than to the purines. If the photophysics of canonical pyrimidines can be predictive, then the longer excited-state lifetime observed for M-Lys compared to melamine suggests that the observed excited state is a ^1^nπ* state. However, this tentative assignment would be strengthened by a future computational study of the positions of the ^1^ππ* and ^1^nπ* states and their associated potential energy surfaces.

## 3. Materials and Methods

### 3.1. TRIR Spectroscopy

The details of the TRIR spectrometer have been described elsewhere [29]. Briefly, the deep UV pump pulses centered at 240 nm were generated from a white light-seeded, two-stage optical parametric amplifier (OPerA Solo, Coherent, Inc., Santa Clara, CA, USA) pumped by approximately 1 W of the 800 nm fundamental (Libra-HE, Coherent; 3.5 W, 80 fs, 1 kHz). The signal beam (NIR) and the fundamental were sum-frequency mixed to generate a 480 nm beam, which was subsequently frequency-doubled to 240 nm. The broadband mid-IR probe pulses were generated from a second optical parametric amplifier (TOPAS + nDFG, Coherent) pumped by approximately 0.8 W of the 800 nm fundamental. The signal and idler beams were then used for difference frequency generation in a GaSe crystal. The resulting mid-IR probe pulses were centered at 6.4 μm (1560 cm^−1^) and had approximately 200 cm^−1^ of bandwidth. The probe beam was split into two portions: the first one was overlapped with the pump and the second one was for a reference.

The pump-probe delay was controlled by a translation stage (Newport, Irvine, CA, USA) that provides a total of 3 ns delay. Every other pump pulse was blocked by a mechanical chopper (New Focus) operating at half of the laser repetition rate. At the sample, the pump pulse energy was attenuated to 2.0 μJ and the spot size was adjusted to 550 μm (fwhm) using a plano-concave CaF_2_ lens. The relative polarization between the electric fields of the pump and probe pulses was set to magic angle (54.7°). The two broadband mid-IR beams were focused into a spectrograph (Triax, Horiba, Irvine, CA, USA), dispersed by a 100 grooves/mm grating, and projected on a dual-row, 64-element/row, N_2_-cooled HgCdTe detector (Infrared Systems Development, Sandscove, FL, USA).

Two mL of solution were flowed through a 100 μm path length flow cell with CaF_2_ windows (Harrick, Pleasantville, New York, NY, USA). The melamine and its lysine derivative were dissolved in D_2_O and the pH of the resulting solutions was adjusted to pD 7.3–7.4 (pD = pH + 0.4) by dropwise addition of DCl or NaOD. D_2_O was used instead of H_2_O for improved IR transparency. The sample concentrations were 10 mM. The unsubstituted melamine (99%) was purchased from Sigma-Aldrich (St. Louis, MO, USA) and used without further purification. The synthesis of M-Lys is described in Section 3.3.

### 3.2. UV-Pump/UV-Probe Transient Absorption (TA) Spectroscopy

The details of the two-color TA (UV-pump/UV-probe) experiments were discussed in Reference [38]. Briefly, the 240 nm pump was generated as described in the previous section. The 350 nm probe was generated by a third optical parametric amplifier (OPerA Solo, Coherent, Santa Clara, CA, USA) pumped by approximately 1 W of the 800 nm fundamental. The signal beam was first frequency-doubled to 700 nm in a BBO crystal. The latter was further frequency-doubled to generate the 350 nm probe beam. 

The probe was optically delayed by a translation stage with respect to the pump. The relative pump-probe polarization was set to magic angle (54.7°). The pump pulse energy and the spot size were adjusted to 0.9 μJ and 350 μm (fwhm), respectively. The probe was focused into a monochromator. The change in the probe in the presence of the pump beam was measured using a photomultiplier tube (PMT) connected to a lock-in detector. The time-resolution of the TA instrument was approximately 480 fs as determined by cross correlation of the 240 nm and 350 nm pulses in the 1 mm cell containing only water. Two mL of solution were flowed through a 1 mm path length flow cell, and the sample concentration was 5 mM.

### 3.3. Synthesis of the Lysine Derivative of Melamine

Boc-Lys-OH (4.94 g, 20 mmol) was stirred with 6-chloro-2,4-diamino-1,3,5-triazine (3.49 g, 24 mmol) and NaOH (1.60 g, 40 mmol) in 35 mL of water at 85 °C overnight. Solid was removed from the solution by centrifuge and the solution was extracted with EtOAc six times. The aqueous solution was then dried under reduced pressure. The Boc protecting group was then removed in 15 mL TFA for 2 h. Most TFA was removed under reduced pressure and to the resulting sticky oil was added cold diethyl ether to precipitate the product. M-Lys was further purified by washing with EtOH. ^1^H NMR (400 MHz, 90% *d*_6_-DMSO + 10% Trifluoroacetic acid-d): δ (ppm) 1.29–1.54 (m, 4H), 1.80 (q, *J* = 8.0 Hz, 2H), 3.26 (t, *J* = 8.0 Hz, 2H), 3.89 (t, *J* = 8.0 Hz, 1H), 7.79–8.71 (m, 2H, acidic protons are suppressed in this acidic NMR solvent and cannot accurately reflect the number of protons). Calculated [M + H] 256.1524; Found [M + H]: 256.1680.

## 4. Conclusions

We have investigated the excited-state dynamics of melamine and its lysine derivative M-Lys. The vast majority of excited melamine molecules decay to the electronic ground state on a time scale of 13 ps after excitation at 240 nm. Photochemical pathways such as intra- and inter-molecular proton transfer and photodissociation are not observed. A small fraction of the excited-state population may be trapped in a long-lived triplet state, but the precise yield is too low to determine reliably given the current signal-to-noise ratio.

The excited-state lifetime of M-Lys is slightly longer than that of the unsubstituted melamine. The change in the excited-state lifetime upon side-chain substitution echoes the behavior of the modern-day pyrimidine nucleobases, which exhibit increased ^1^nπ* state lifetimes in the presence of ribose and phosphoribose substituents. Overall, the fast dynamics observed for melamine, an important *s*-triazine derivative and possible prebiotic alternative nucleobase, are consistent with a high degree of hardiness to UV radiation.
